# Identification of new sources of resistance to charcoal rot caused by *Macrophomina phaseolina* (Tassi) Goid in cowpea

**DOI:** 10.1186/s12870-025-06628-1

**Published:** 2025-05-17

**Authors:** Francis Sodji, Theophilus Kwabla Tengey, Charles Kodia Kwoseh

**Affiliations:** 1 Department of Crop and Soil Sciences, Kwame Nkrumah University of Science and Technology, Kumasi, Ghana; 2 Council for Scientific and Industrial Research-Savanna Agricultural Research Institute (CSIR-SARI), Nyankpala, NL-1032-0471 Ghana

**Keywords:** Disease incidence, Disease severity, Host-plant resistance, Inoculation, Pathogen

## Abstract

Charcoal rot caused by *Macrophomina phaseolina* results in substantial yield losses in cowpea. Host plant resistance is the most reliable and cost-effective method of controlling this disease. Newly developed varieties need to be screened for their reaction to *M. phaseolina* as new strains of the pathogen keep evolving due to pathogen reproduction. This study sought to identify cowpea genotypes with stable resistance to *M. phaseolina*. Twenty-three cowpea genotypes were screened on the field and in pots under screenhouse conditions using a virulent *M. phaseolina* isolate. The pathogen effect on the genotypes and their agronomic performance and yield were determined. Disease parameters such as percentage Disease Incidence (DI) at the seedling stage, Seedling Infection Score (SIS), Root and Stem Severity (RSS), and Colony Forming Unit Index (CFUI) measured on the field were highly correlated with screen house experiments. These parameters were also significantly and positively correlated with each other suggesting that any of these parameters could be used in ranking the resistance or susceptibility of the genotypes. The disease parameters also had a significant negative correlation with percent germination/plant stand and pod yield and grain yield. It was observed that the charcoal rot disease was more severe at the seedling stage than at the reproductive stage. This is evidenced by the significant strong positive correlation between percent germination/plant stand and, pod yield and grain yield as the disease at the seedling stage causes seed rot and damping off. Based on the most Frequent Genotype Rank (FGR) generated by recording the highest occurring genotype rank of SIS, RSS, and CFUI of each genotype and comparison with grain yield data, IT11K-61-82, IT10K-837-1, SARI-3-11-100 and IT84S-2049 were ranked to be resistant to moderately resistant while IT07K-303-1, IT14K-1682-3 and IT13K-1070-2 were moderately susceptible. Additionally, IT14K-2030-2 and IT14K-2113-4 were susceptible to Charcoal rot disease while the remaining were moderately susceptible. Genotypes that combine resistance with farmer-preferred agronomic traits should be released as varieties while those that lack the farmer-preferred traits could be improved through hybridizations.

## Introduction

Cowpea (*Vigna unguiculata* (L.) Walp), as a grain legume crop is an important source of food, income and livestock feed and forms a major component of tropical farming systems because of its ability to improve marginal lands through nitrogen fixation and as cover crop. The grain is also a good source of protein for humans, while the haulm is important for livestock [15]. Many smallholder farmers and traders in Sub-Saharan Africa depend on it as a valuable and stable source of income [21]. Cowpea's deep roots can help to improve soil structure, and its fast growth and soil coverage can help to reduce erosion [13].

Approximately 80% of the cowpea produced in Ghana is grain, largely in the savannah zones [14]. Even though cowpea has a wide range of seed yields, yields are the lowest in the world, with an average of 310 kg/ha [29]. Cowpea faces numerous production constraints despite its importance. In Ghana, pests and diseases, low soil fertility, as well as drought, are the major constraints of cowpea production [19].

*Macrophomina phaseolina* (Tassi) Goid is the causal agent of charcoal rot, a polyphagous soil-borne pathogen that infects more than 500 different plant species [6, 7, 40, 43]. Root rot, stem rot, damping off, wilt, leaf blight, stem blight, seed rot, and pod rot are among the diseases caused by *M. phaseolina* infection [34]. The most common disease caused by this pathogen is charcoal rot [37]. The main source of inoculum for new infections is micro-sclerotia in soil and plant debris [20]. Drought stress affects the physiology of the host plant, weakening plant tissues and making crops more vulnerable to facultative *M. phaseolina* [22]. *Macrophomina phaseolina* has been found in bean-growing areas in West Africa, where the weather is hot [11]. This fungus can live in the soil as sclerotia embedded in organic debris or free in the soil [1, 33, 39]. Host crops exhibit a wide range of disease symptoms, which can occur at any stage of development. In seedlings, *M. phaseolina* can cause pre- or post-emergence damping off, black cotyledonary lesions at varying degrees of severity or it can persist in a crop showing little to no disease symptoms. Apart from having a wide host range, the fungus produces sclerotia that remain viable in the soil for many years, and it has been difficult to find suitable sources of resistance genes among cowpea genotypes [8]. When host plants are attacked by *M. phaseolina*, severe yield losses may occur. In the USA, charcoal rot has resulted in about 65% yield loss in common bean growing areas [25]. In many bean-growing locations in Nigeria, outbreaks of epidemic proportions have occurred, with production losses ranging from 46 to 74% due to charcoal rot of cowpea caused by *M. phaseolina* [30].

In the event of trying to prevent infection by *M. phaseolina,* various control measures have been employed such as biological, cultural and chemical control practices [30]. In Africa where cowpea is mostly grown by small-scale farmers, these control strategies have not proven to be effective [23, 30]. Chemical soil fumigation, for example, is prohibitively costly for the average farmer. As a result, majority of the effort has gone into creating long-term control solutions. While efforts have been made to breed *M. phaseolina*-resistant genotypes, little research has been done on screening several common cowpea cultivars for resistance. High-yielding cowpea cultivars are susceptible to *M. phaseolina*-caused diseases, especially charcoal rot [17]. Particularly in West Africa, exotic cowpea cultivars that are high yielding have proved to be very susceptible to this devastating pathogen (Mughogho and Pande, 1983). Songotra (IT99K-499-35), an early maturing, high yielding variety released by the CSIR-SARI in 2008, has been withdrawn in Ghana due to *M. phaseolina* susceptibility [9].

In Ghana, efforts have been made to improve cowpea production through various means, including the introduction of new varieties [3]. Identifying cowpea genotypes resistant to charcoal (ashy) rot appears promising because host resistance may be the only viable method for control, reducing pathogen damage and yield losses, and may serve as additional sources of charcoal rot resistance in breeding programmes. Therefore, *Macrophomina*-resistant varieties of cowpea are important because they will help reduce damage and increase yield for a sustainable cowpea production. The main objective of this study was to determine the effect of *M. phaseolina* on grain yield of cowpea. The specific objectives were to; i) determine the reaction of the cowpea genotypes to the most virulent *M. phaseolina* isolate under screen house and field conditions, ii) classify the genotypes based on their level of resistance to charcoal rot (*M. phaseolina*), and iii) identify promising cowpea genotypes that are high yielding and also resistant to *M. phaseolina*.

## Materials and methods

### Location of experiment

The experiment was carried out at the Plant Pathology and Nematology Laboratory and experimental fields of the CSIR-Savanna Agricultural Research Institute (SARI), Nyankpala-Tamale. The area is situated at latitude 9° 25"N and longitude 1° 00"W, 183 m above sea level, with total annual rainfall of 1000–1300 mm per annum.

### Plant materials and their source

A total of 23 genotypes of cowpea (Table 1) were evaluated. These include 18 advanced cowpea breeding lines (genotype) and five checks. The advanced breeding lines originates from crosses with diverse parents and are above the F_7_ stage and are therefore homozygotes. The susceptible checks include, IT97K-499-35 released in Ghana as Songotra and original IT97 K-499-35 obtained from International Institute of Tropical Agriculture (IITA). The resistant checks were Apaagbala, SUVITA-2 and IT99K-573-1-1. As a result of limited seed quantity, all the five checks were used in the screenhouse study and only three (Songotra, IT97K-499-35 and Apaagbala) were evaluated under field conditions.

**Table 1:** List of advanced cowpea breeding genotypes and checks used for the studies

No	Genotype	Pedigree	Botanical status	Source
1	IT10K-837-1	IT97K-499-35 x IT99K-241-2	Advanced breeding line	IITA
2	KVX404-8-1x693-2		Advanced breeding line	INERA
3	IT13K-1070-2	IT99K-573-2-1 x IT97K-499-35	Advanced breeding line	IITA
4	IT13K-1144-2		Advanced breeding line	IITA
5	IT10K-817-3	IT03K-337-6 x IT97K-1042-3	Advanced breeding line	IITA
6	IT84S-2049		Advanced breeding line	IITA
7	IT07K-303-1	IT89D-245 x IT95K-1072-57	Advanced breeding line	IITA
8	IT14K-2113-4		Advanced breeding line	IITA
9	IT14K-1682-3		Advanced breeding line	IITA
10	SARI-3-11-80	Padi-tuya x Sanzi	Advanced breeding line	CSIR-SARI
11	IT14K-1424-12	IT90K-372-1-2 x IT97K-499-38	Advanced breeding line	IITA
12	SARI-2-50-80	Padi-tuya x Sanzi	Advanced breeding line	CSIR-SARI
13	SARI-3-11-100	Padi-tuya x Sanzi	Advanced breeding line	CSIR-SARI
14	IT11K-61-82		Advanced breeding line	IITA
15	IT14K-1850-2		Advanced breeding line	IITA
16	SARI-6-2-6	Padi-tuya x Sanzi	Advanced breeding line	CSIR-SARI
17	IT14K-2030-2		Advanced breeding line	IITA
18	Padi-tuya		Released variety	CSIR-SARI
19	Apaagbala	(IT82E-16 x Prima) x 148-1	Released variety	CSIR-SARI
20	Songotra		Released variety	CSIR-SARI
21	IT97K-499-35		Released variety	IITA
22	IT99K-573-1-1		Released variety	CSIR-SARI
23	SUVITA-2		Advanced breeding line	CSIR-SARI

*NB: CSIR-SARI*: Council for Scientific and Industrial Research-Savanna Agricultural Research Institute, *IITA*: International Institute of Tropical Agriculture, *INERA*: Institute of Environment and Agricultural Research

### Source of *M. phaseolina* and preparation of inoculum

A seven-day-old culture of *M. phaseolina* isolate (Mp_3Mc) previously reported to be the most virulent isolate [38] was used. The rice method of inoculation described by Schoonhoven and Pastor-Corrales [36] with modification by autoclaving 100 g of rice seeds in a 1L flask containing 10 mL of distilled water was used to inoculate the test plants. In this method, rice grain were soaked with distilled water, autoclaved at a temperature of 121 °C at a pressure of 0.98 kg/cm^2^ for 15 min and allowed to cool. The isolate was cut using a 10 mm cork borer at the margin to obtain mycelial discs. Five mycelial discs were placed on top of the autoclaved rice grain and kept in the dark for about 14 days to allow proper growth of the fungus on the rice grain. Test plants were inoculated using the dried inocula of the most virulent *M. phaseolina* isolate described above.

### Sterilization of soil for pot experiment

A pot mixture was made of 70% loam and 30% sand were thoroughly mixed and steam sterilized as described by Awuku [10]. The pot mixture is placed on top of a jute sack laid in a mesh in a barrel containing 1/3 volume of water. Steam rising from water in the barrel through the soil sterilizes it. The barrel was covered to ensure effective sterilization. The sterilized soil was allowed to cool after which it was filled in pots with diameter 30 cm and height of 30 cm. Pots have drainage holes at the bottom to prevent waterlogging.

### Pot experiment in the screenhouse

Two experiments were conducted under screenhouse conditions. These included seedling stage screening and reproductive stage screening.

#### Screening of seedlings of cowpea genotypes

Two separate experiments were conducted, with the first experiment having four (4) replications while the second experiment had three (3) replicatons with the 23 genotypes arranged in a Complete Randomized Design. Screening was done in pots as described above. A single seed of each genotype was planted per pot alongside inocula of three rice-infected grain (inoculum) to initiate infection. Pots were watered below the test plants once daily to maintain soil moisture content. Rotted seeds, pre- and post- emergence damping off were rated at 10 days and aboveground infection of seedlings was assessed at 20 days after planting according to an adapted *M. phaseolina* rating scale provided by Abawi and Pastor-Corrales [2] for common bean. Data were collected by scoring symptoms of the disease using a modified scale (1–9) of Abawi and Pastor-corrales [2] for common bean adopted for cowpea as follows. A score of 10 was included to cater for seed rot and damping off. This visual scale was used based on disease severity exhibited below the cotyledon node for the seedlings infection score.

**Table:** 

Scale	Description
1	No visible symptoms.
3	Lesions are limited to cotyledonary tissues.
5	Lesions have progressed from cotyledons to about 2cm of stem tissues.
7	Lesions are extensive on stem and branches. Chlorosis and necrosis on foliage.
9	Most of the stem, petioles, and growing point are infected.
10	Seed rot, pre and post-emergence damping off.

A score of 10 was included to account for viable seeds that got rotten, pre- and post- emergence seedlings that died due to the effect of the pathogen [18].

#### Screening of reproductive stage of cowpea genotypes

With the reproductive stage screening, inoculation was done at the unifoliate stage (14 days after planting). Soil around the root zones of the test plants was carefully removed and three grains of infected rice were buried around the root zone placing them tightly to the lateral roots. Pots were watered once daily to maintain a soil moisture content of 16% and temperature 32 °C. Soil moisture was measured using soil moisture meter. At first pod maturity (R7, a single pod exhibiting mature colour), the test plants were harvested by uprooting them. Prior to harvesting, sides of the plastic pots were pressed to loosen the soil to ensure easy removal of the plants from the soil. The soils were then removed from the roots by cautiously shaking the plants. The protocols for field plot disease assessment were used as described by Mengistu et al. [24], which involved measuring root and stem severity (RSS) and determining the colony-forming units (CFU).

### Verification of *M. phaseolina*

Rotted seeds, pre- and post- emergence damping off and infected seedlings displaying various stages of disease were collected for verification of *M. phaseolina*. Diseased root and stem samples, excluding rotted seeds, were cut into pieces and disinfected in a 1% ethanol-sodium hypochlorite solution for 1 min, then rinsed in sterile distilled water for an additional minute. The samples were placed on PDA plates, allowing fungal mycelium to grow for seven days. Once established, a heat-sterilized inoculating needle was used to transfer *M. phaseolina* hyphae onto new PDA plates, which were then incubated at room temperature (29 ± 1 °C). The identification procedure described by Gupta et al. [16] was used to identify the fungus, where the pathogen produced dark sclerotia when viewed under compound microscope.

### Screening of cowpea genotypes under field conditions

#### Location of experiment

Field plots were established at the CSIR-Savanna Agricultural Research Institute (SARI) at Nyankpala,Tamale. A known naturally infected field with *M. phaseolina* was used for the trial.

#### Field layout and experimental design

The experiment was laid out in a randomized complete block design (RCBD) with three replications. The treatment included 21 cowpea genotypes. Each plot was a 2 m-double row with an inter-row spacing of 0.6 m and intra-row spacing of 0.2 m and sowing depth of about 2 cm. Plots were separated by 1 m alley with 1.5 m alleys separating replicates. The experiment was conducted in the dry season (October, 2020 to January 2021) as the pathogen thrives best in this season. The average monthly temperature from October to January were 32.5 °C, 36.4 °C, 37.2 °C, and 37.0 °C, while the average monthly rainfall during the same period were 133.9 mm, 0.0 mm, 0.0 mm, and 0.0 mm, respectively.

#### Planting, field inoculation, agronomic practices and harvesting

Field selected for screening is known to have charcoal rot disease occurrence over the years with soil type being sandy-loam. Isolates of *M. phaseolina* were obtained from this field and were found to be virulent [38]. The field was sprayed with Force-up (360 g Glyphosate/L) weeks prior to ploughing to kill existing weeds. After harrowing, a field layout was made. Two seeds were planted at stake per hill. In order to reduce plot-to-plot variability, the genotypes were further inoculated with six-rice grains infected with *M. phaseolina* per hill at the time of planting using the inoculation method described above. Basal application of NPK 15–15-15 was done to avoid nitrogen and phosphorus deficiencies. Side placement of NPK was done two weeks after planting. Three insecticide applications using manufacture’s recommendation were done before flowering, during flowering and pod developments to control insect pests. The insecticide used was Power (Lamda, cyahalothrin and dimethoate). Fields were maintained weed-free by weeding as at when necessary. Plots were irrigated at four days interval. At the first pod maturity (R7), the test plants were harvested by uprooting for assessment of root and stem severity. Prior to this fields were irrigated to moisten soil around test plants. Data was collected on days to first flowering (DFF), days to 50% flowering (D50F), days to first pod maturity (D1PM), days to 95% pod maturity (D95PM), pod yield (PY), grain yield (GY), dry biomass yield (BDW) and harvest index (HI).

### Assessment of seedlings infection

In the field and screenhouse setups that were used to screen for *Macrophomina* resistance at the seedling stage, data were collected by scoring symptoms of the disease using a modified scale (1–9) of Abawi and Pastor-corrales [2] for common bean adopted for cowpea.

Stand density was evaluated at two weeks after planting by counting germinated plants in each plot. Disease incidence and severity at seedling stage were assessed. Disease incidence was assessed by counting diseased plants per total number of plants in each plot and expressed in percentage whiles disease severity was assessed using Abawi and Pastor-Corrales [2] 1–9 scale with modification by including a score of 10 as described above. Aboveground infection of seedlings was assessed at 21 days after planting and were rated according to a modified *M. phaseolina* rating scale by Abawi and Pastor-Corrales [2]. This visual scale was used based on disease severity exhibited below the cotyledon node. Genotypes were subsequently ranked as resistant (1 to 3), moderately resistant (values 4 to 5), moderately susceptible (values 6 to 7) and susceptible (values of 8 to 10).

### Preparation of harvested plants and data collection

At the R7, five individual plants were randomly sampled from each plot and axillary roots and branches were removed. The stems, which included the taproots were thoroughly washed off excess soil, bundled and placed in envelop bags to air dry and stored at room temperature. This period allowed any existing microsclerotia to develop within the root-stem system and for drying so that the roots and stems can be blended to a finer powder before plating to determine the CFU’s.

Field plot disease assessment protocols were used according to Mengistu et al. [24]. This includes determination of root and stem severity (RSS) and colony forming unit (CFU).

#### Assessment of root and stem severity

Root and stem severity (RSS) was assessed by longitudinally splitting each plant's stem and taproot and visually rating the intensity of discolouration caused by microsclerotia growth within the root-stem system. The ratings were given on a scale of 1 to 5, with 1 denoting no discolouration and 5 denoting extreme discoloration [24, 32].

In the field and screenhouse setups that were used to screen for *Macrophomina* resistance at the reproductive stage, root and stem severity (RSS) was assessed by visually rating the intensity of discolouration from microsclerotia development in split lower stem and root sections of each plant using a scale of 1–5 rating system adopted from Mengistu et al. [24] and Paris et al. [32] as follows,

**Table:** 

Scale	Description
1	no apparent microsclerotia in the tissue
2	relatively few microsclerotia visible in the pith, vascular tissue, or underneath the epidermis, and the vascular tissue has not been discoloured
3	microsclerotia have partially covered the tissue and the vascular tissue is partly discoloured
4	microsclerotia are apparent under the outer epidermis in stem and root sections, and vascular tissue is discoloured with many microsclerotia embedded in tissue
5	vascular tissue darkened due to high numbers of microsclerotia both inside and outside of the stem and root tissues

Mengistu et al. [24] categorized cultivar resistance or susceptibility using RSS scale scores: resistant (values of 1), moderately resistant (values > 1 and ≤ 2), moderately susceptible (values > 2 and < 3) and susceptible (values of 3 to 5).

#### Determination of colony forming units

The five plant samples per plot used for the RSS assessment after splitting were also used to determine the amount of *M. phaseolina* colony-forming units (CFU) present in the stem. Samples were composited and cut into pieces and surface sterilized using 10% sodium hypochlorite (bleach) for three minutes, followed by 70% ethanol for 3 min and then rinsed in three changes of sterilized distilled water, each for 3 min. These were then dabbed dry with paper towel and allowed to air-dry. Samples of 5 g each were blended separately with a laboratory blender at 1 min interval for three times and sieved using 1 mm mesh-screen. The blender and mesh-screen were thoroughly cleaned between each sample to avoid sample-to-sample contamination. One gramme (1 g) each of the sieved samples were then used to prepare twofold serial dilution (10^–2^). One mililitre (1 mL) was pipetted and dispensed onto PDA Petri plates and swirlled gently. The plates were incubated at the incubation room for three days at room temperature. The number of *M. phaseolina* colonies on each plate was counted, and the results converted to CFUg^−1^ tissue, by dividing each genotypes's CFUg^−1^ with the highest average CFUg^−1^ of the susceptible cultivar within the experimental plot, a colony forming unit index (CFUI) was determined [24]. Since ITK97K-499–35 had the highest average CFUg^−1^, all CFUI data were based on CFU from this genotype. The genotypes were then categorized as resistant (0 to < 10), moderately resistant (10 to ≤ 30), moderately susceptible (> 30 to 60) and susceptible (> 60) based on CFUI, according to Schmitt and Shannon's [35] classification scheme for Soybean and Cyst Nematode (SCN) adapted for *Macrophomina* by Mengistu et al. [24]. The CFUI serves as a quantifiable measure of pathogen load in the host plant. For a non-spore forming pathogen such as *Macrophomina*, which forms sclerotia, the CFUI provides insight into the concentration and proliferation of sclerotia within the host tissues. For genotypes claimed to be resistant, CFUI provides a measurable parameter to validate these claims. Lower CFUI values in resistant genotypes confirm that these plants are less conducive to pathogen proliferation, thereby validating the genotype's resistance.

### Ranking of genotypes

Genotypes from field and screenhouse experiments were first ranked as resistant (R), moderately resistant (MR), moderately suceptible (MS) and susceptible (S) based on the defined categories described by Abawi and Pastor-Corrales [2] for Seedling infection stage (SIS), Mengistu et al. [24] for root and stem severity (RSS) and Schmitt and Shannon's [35] classification scheme for soybean and cyst nematode (SCN) adapted for *Macrophomina* by Mengistu et al. [24] for colony forming unit index (CFUI). In order to give an overall ranking of genotypes from field and screenhouse experiments for SIS, RSS and CFUI, the most frequently occurring genotype rank (FGR) for each genotype was observed and recorded as the final resistance category of the genotype.

### Data analysis

Data were analyzed using GENSTAT statistical package (12^th^ edition) software. Means were separated using LSD at 5%. Correlation analysis was done using the R-software.

## Results

**Fig. 1:**
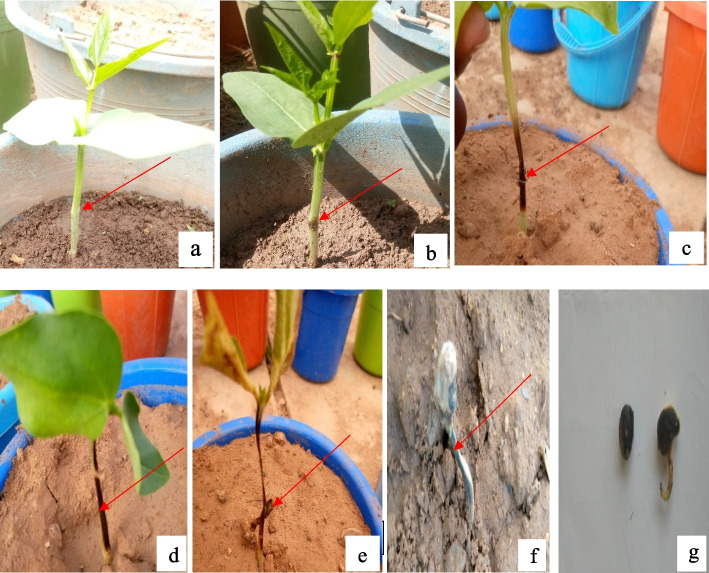
Symptoms of *M. phaseolina *infection. **a** disease score of 1. **b** disease score of 3. **c** disease score 5. **d** disease score of 7. **e** disease score of 9. **f** disease score of 10. **g** disease score of 10

Symptoms of charcoal rot disease at the seedling stage and their scores have been shown in Fig. 1. The overall seedling infection score from the two separate experiments at the seedling stage of the genotypes at 21 days after sowing and inoculation ranged from 1.56 to 8.39 (Table 2). The lowest infection score (1.56) was observed in Apaagbala which was not significantly different (*p* > 0.05) from other resistant and moderately resistant genotypes such as IT11K-61–82, SARI-3-11-100, SUVITA-2, IT10K-837-1, and IT84S-2049 in expressing the charcoal rot disease. However, IT14K-2030-2 and IT14K-2113-4, exhibited the highest infection of the pathogen with scores of 8.39 and 8.28 respectively. The rest of the genotypes particularly, IT07K-303-1, IT14K-1682-3, and IT13K-1070-2, had scores either equal to the susceptible check or greater but were not significantly different (*p* > 0.05) from the susceptible check.

**Table 2:** Seedling infection of cowpea genotype under screenhouse conditions

	Seedling Infection Score (SIS)		
	Experiment 1	Experiment 2	Experiment 1 & 2
IT07K-303-1	6.00	6.33	6.00
IT10K-817-3	7.25	6.33	6.72
IT10K-837-1	2.50	2.33	2.56
IT11K-61-82	1.50	1.67	1.67
IT13K-1070-2	7.50	7.33	7.50
IT13K-1144-2	5.75	5.67	5.90
IT14K-1424-12	7.50	7.67	7.56
IT14K-1682-3	6.75	7.00	6.89
IT14K-1850-2	7.00	7.67	7.33
IT14K-2030-2	8.25	8.33	8.39
IT14K-2113-4	8.50	8.00	8.28
IT84S-2049	2.50	2.33	2.33
KVX404-8-1x693-2	5.50	5.67	5.44
SARI-2-50-80	7.50	7.67	7.44
SARI-3–11−100	2.00	2.33	2.11
SARI-3-11-80	6.50	6.33	6.44
SARI-6-2-6	5.50	5.67	5.67
SUVITA-2	2.50	2.33	2.56
IT99 K-573-1-1	5.00	5.00	4.78
Padituya	8.50	6.33	7.56
Apaagbala	1.50	1.67	1.56
Songotra	6.50	7.67	7.00
IT97K-499-35	6.50	6.33	6.33
LSD (0.05)	2.52	2.63	1.85
CV	32.0	28.8	20.2

SIS score (1–10), *SIS *Seedling Infection Score

The genotypes exhibited varying degrees of susceptibility to the pathogen, which was reflected in their RSS and CFUI values. There was significant differences (*p* < 0.05) in the mean Root and Stem severity (RSS) likewise CFUI values of the genotypes taken five days after harvest. The RSS ranged from 1.25 in Apaagbala to 4.50 in IT97K-499-35 (Table 3). Genotypes categorized as moderately resistant to resistant include IT11K-61-82, IT10K-837-1, SARI-3-11-100, and IT84S-2049. These genotypes also had lower RSS and CFUI values against the pathogen. Resistant genotypes such as IT10K-837-1 and IT84S-2049 had low RSS values of 1.50 and 1.75 and low CFUI values of 9.80 and 13.91, respectively while susceptible genotypes such as IT14K-2030-2 and IT14K-2113-4 exhibited higher RSS of 4.25 and 3.75 and a higher CFUI of 68.75 and 57.28 respectively (Table 3).

**Table 3:** Cowpea genotype reaction to *M. phaseolina* at the reproductive stage under screenhouse conditions

Cowpea genotypes		RSS	CFU	CFUI
IT07K-303-1		3.75	24.00	49.39
IT10K-817-3		3.50	22.50	46.27
IT10K-837-1		1.50	4.75	9.80
IT11K-61–82		1.75	6.75	14.10
IT13K-1070-2		3.50	27.50	56.75
IT13K-1144-2		3.25	22.00	45.33
IT14K-1424-12		3.25	23.50	37.21
IT14K-1682-3		2.75	19.25	39.67
IT14K-1850-2		3.25	21.75	44.99
IT14K-2030-2		4.25	33.25	68.75
IT14K-2113-4		3.75	27.75	57.28
IT84S-2049		1.75	6.75	13.91
KVX404-8-1x693-2		3.25	26.25	54.16
SARI-2-50-80		3.25	23.00	47.48
SARI-3-11-100		1.75	8.00	16.55
SARI-3-11-80		3.00	21.00	43.34
SARI-6-2-6		2.50	17.75	36.66
SUVITA-2		2.00	9.75	20.12
IT99K-573-1-1		2.50	15.25	31.42
Padi-tuya		3.00	16.00	33.11
Apaagbala		1.25	2.88	5.80
Songotra		3.50	33.50	69.21
IT97K-499-35		4.50	46.75	96.42
LSD (0.05)		0.7736	5.787	12.72
CV (%)		18.90	20.50	22.10

*RSS *Root and stem severity, *CFU *Colony forming unit, *CFUI *Colony forming unit index

The study evaluated the performance of various cowpea genotypes in response to *M. phaseolina* infection, considering both disease parameters such as Disease Incidence (DI), Root and Stem Severity (RSS), and Colony Forming Unit Index (CFUI), as well as percentage germination or plant stands. Based on these metrics, the genotypes were categorized into resistant, tolerant, susceptible, and moderately susceptible groups. Genotypes IT11K-61-82, IT10 K-837-1, SARI-3-11-100, and IT84S-2049 demonstrated strong resistance to *M. phaseolina* infection (Table 4). These genotypes exhibited significantly lower DI, RSS, and CFUI values, coupled with high plant stands. For instance, IT11K-61-82 had a DI of 7.75%, RSS of 1.77, CFUI of 11.56, and a high plant stands of 74.17%. Similarly, IT84S-2049, with the lowest DI of 6.13%, had an RSS of 2.00, CFUI of 12.25, and a high germination rate of 69.17% (Table 4).

**Table 4:** Effect of *M. phaseolina* on percentage germination, disease incidence, seedling inflection and reproductive stage of cowpea genotypes under field conditions

Cowpea genotypes	Percentage germination	%DI at seedling stage	SIS	RSS	CFUI
IT07K-303-1	56.67	15.36	5.33	3.33	44.90
IT10K-817-3	39.17	39.33	7.44	3.07	41.49
IT10K-837-1	67.50	7.22	4.16	1.97	8.84
IT11K-61–82	74.17	7.75	3.64	1.77	11.56
IT13K-1070-2	36.67	27.99	7.23	3.37	48.98
IT13K-1144-2	40.00	21.37	6.88	3.43	48.12
IT14K-1424-12	43.33	28.23	6.70	3.23	61.91
IT14K-1682-3	45.83	22.11	6.383	2.37	40.14
IT14K-1850-2	38.33	20.22	7.00	2.97	51.70
IT14K-2030-2	37.50	30.26	7.43	3.23	71.43
IT14K-2113-4	40.83	27.49	6.96	3.73	62.58
IT84S-2049	69.17	6.13	3.88	2.00	12.25
KVX404-8-1x693-2	41.67	27.12	6.97	3.07	53.74
SARI-2-50-80	44.17	16.75	6.41	2.87	49.50
SARI-3-11-100	63.33	8.91	4.65	2.20	17.01
SARI-3-11-80	44.17	26.15	6.63	3.00	36.05
SARI-6-2-6	42.50	20.09	6.64	2.53	27.89
Padi-tuya	39.17	19.56	6.82	2.63	28.57
Apagbaala	76.67	4.30	3.23	1.50	8.16
Songotra	45.83	27.27	6.61	3.50	70.75
IT97K-499-35	31.67	32.57	8.05	4.33	100.00
LSD (0.05)	12.244	11.82	1.13	0.66	11.47
CV (%)	15.30	34.50	11.20	14.00	16.30

*% DI *Percentage disease incidence at seedling stage, *SIS *Seedling infection score (severity), *RSS *Root and stem severity, *CFUI *Colony forming unit index

However, genotypes IT14K-2030-2 and IT14K-2113-4 were identified as highly susceptible to *M. phaseolina*, showing high DI, RSS, CFUI values, and lower germination percentages. IT14K-2030-2 had a DI of 30.26%, RSS of 3.23, the highest CFUI of 71.43, and a lower plant stands of 37.50%. Similarly, IT14K-2113-4 had a DI of 27.49%, RSS of 3.73, CFUI of 62.58, and a plant stand of 40.83%. Conversely, genotypes such as IT07K-303-1, IT14K-1682-3, and IT13K-1070–2 were classified as tolerant because, despite higher disease incidence and severity, they maintained moderate germination rate and plant stands. IT07K-303-1 had a DI of 15.36%, RSS of 3.33, CFUI of 44.90, and a plant stands of 56.67%. Similarly, IT14 K-1682–3 had a DI of 22.11%, RSS of 2.37, CFUI of 40.14, and a germination rate of 45.83% (Table 4).

**Fig. 2:**
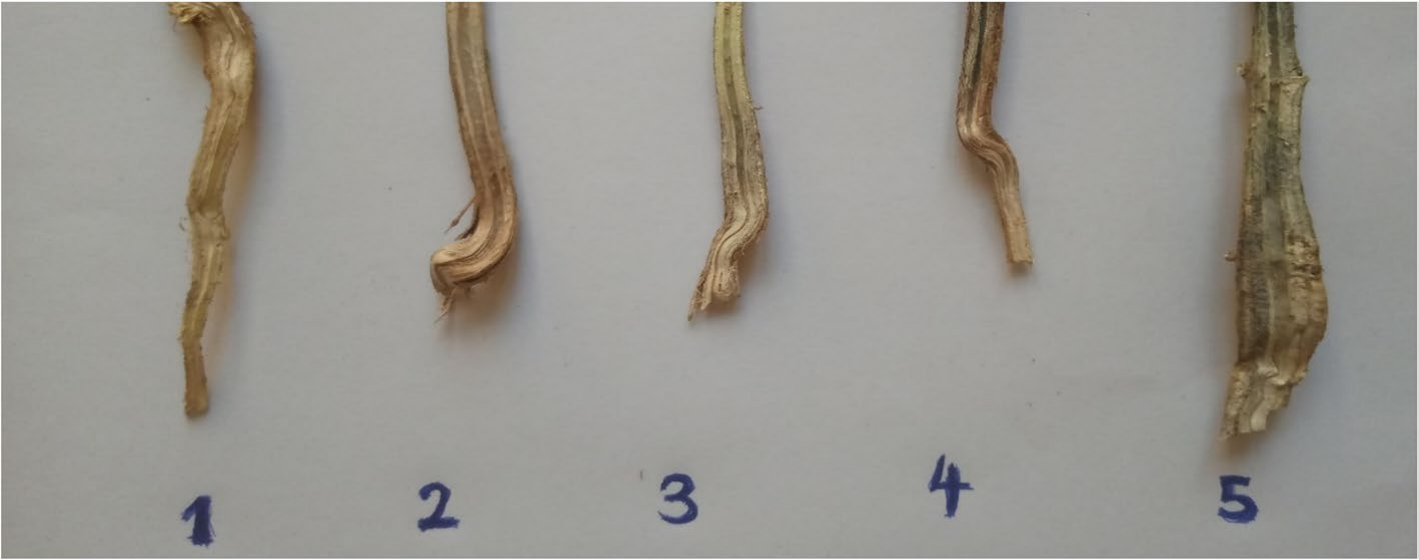
Evaluation for root and stem severity (RSS) in split lower stem and root sections showing *M. phaseolina* microsclerotia on scale of 1–5 (rating system adopted from [32])

The remaining genotypes, such as Songotra, KVX404-8-1 × 693-2 and IT14K-1424-12, were categorized as moderately susceptible, displaying high levels of disease indicators and variable germination percentages. The RSS of genotypes were classified as shown in Fig. 2.

The mean CFUI (%) of the genotypes after harvest ranged from 8.84% to 100.00%. Apaagbala had the least CFUI (8.84%) and IT97K-499-35 had the highest (100.00%). According to Schmitt and Shannon's [35] classification scheme for SCN [24], Apaagbala and IT10K-837-1 had CFUI values < 10. IT84S-2049, SARI-3-11-100, SARI-6-2-6 and Padituya recorded CFUI percentages > 10 < 30. CFUI values > 30 < 60 were observed between IT14K-1682-3, IT10K-817-3, IT07 K-303-1, IT13K-1144-2, IT13K-1070-2, SARI-2-50-80, IT14K-1850-2 and KVX404-8-1 × 693-2. CFUI values > 60 were recorded among IT14K-1424-12, IT14 K-2113–4, Songotra, IT14K-2030-2 and IT97K-499-35. A summary of resistance or susceptibility of cowpea genotypes to *M. phaseolina* screened under pot and field conditions have been provided in Table 5.

**Table 5:** Summary of resistance or susceptibility of cowpea genotypes to *M. phaseolina* screened under pot and field conditions

	Screen house reaction							Field reaction							
Genotypes	SIS	Rxn	RSS	Rxn	CFUI	Rxn	FGR	SIS	Rxn	RSS	Rxn	CFUI	Rxn	FGR	Overall FGR
IT07K-303-1	6.00	MS	3.75	S	49.39	MS	MS	5.33	MR	3.33	S	44.90	MS	MS	MS
IT10K-817-3	6.72	MS	3.50	S	46.27	MS	MS	7.44	MS	3.07	S	41.49	MS	MS	MS
IT10K-837-1	2.56	R	1.50	MR	9.80	R	R	4.16	MR	1.97	MR	8.84	R	MR	MR/R
IT11K-61–82	1.67	R	1.75	MR	14.10	MR	MR	3.64	R	1.77	MR	11.56	MR	MR	MR
IT13K-1070-2	7.50	MS	3.50	S	56.75	MS	MS	7.23	MS	3.37	S	48.98	MS	MS	MS
IT13K-1144-2	5.90	MR	3.25	S	45.33	MS	MS	6.88	MS	3.43	S	48.12	MS	MS	MS
IT14K-1424-12	7.56	MS	3.25	S	37.21	MS	MS	6.70	MS	3.23	S	61.91	S	S	MS/S
IT14K-1682-3	6.98	MS	2.75	MS	39.67	MS	MS	6.38	MS	2.37	MS	40.14	MS	MS	MS
IT14K-1850-2	7.33	MS	3.25	S	44.99	MS	MS	7.00	MS	2.97	MS	51.70	MS	MS	MS
IT14K-2030-2	8.39	S	4.25	S	68.75	S	S	7.43	MS	3.23	S	71.43	S	S	S
IT14K-2113-4	8.28	S	3.75	S	57.28	MS	S	6.96	MS	3.73	S	62.58	S	S	S
IT84S-2049	2.33	R	1.75	MR	13.91	MR	MR	3.88	R	2.00	MR	12.25	MR	MR	MR
KVX404-8-1x693-2	5.44	MR	3.25	S	54.16	MS	MS	6.97	MS	3.07	S	53.74	MS	MS	MS
SARI-2-50-80	7.44	MS	3.25	S	47.48	MS	MS	6.41	MS	2.87	MS	49.50	MS	MS	MS
SARI-3–11-100	2.11	R	1.75	MR	16.55	MR	MR	4.65	MR	2.2	MS	17.01	MR	MR	MR
SARI-3-11-80	6.44	MS	3.00	S	43.34	MS	MS	6.63	MS	3.0	S	36.05	MS	MS	MS
SARI-6-2-6	5.67	MR	2.50	MS	36.66	MS	MS	6.64	MS	2.53	MS	27.89	MR	MS	MS
SUVITA-2	2.56	R	2.00	MR	20.12	MR	MR	-	-	-	-	-	-		MR
IT99K-573-1−1	4.78	MR	2.50	MS	31.42	MS	MS	-	-	-	-	-	-		MS
Padituya	7.56	MS	3.00	MR	33.11	MS	MS	6.82	MS	2.63	MS	28.57	MR	MS	MS
Apaagbala	1.56	R	1.25	MR	5.80	R	R	3.23	R	1.50	MR	8.16	R	R	R
Songotra	7.0	MS	3.50	S	69.21	S	S	6.61	MS	3.50	S	70.75	S	S	S
IT97K-499-35	6.33	MS	4.50	S	96.42	S	S	8.05	S	4.33	S	100	S	S	S

*Rxn *Reaction, *R *Resistance, *MS *Moderately susceptible, *S *Susceptible, *FGR *Frequent genotype rank

Disease parameters measured based on SIS, RSS, CFU, CFUI for cowpea genotypes tested under field conditions and screenhouse conditions were positively and strongly correlated (Table 6).

**Table 6:** Coefficient of correlation between disease parameters of tested genotypes under screenhouse and field conditions

	SISs	RSSs	CFUs	CFUIs	SISf	RSSf	CFUf
SISs							
RSSs	0.88***						
CFUs	0.83***	0.92***					
CFUIs	0.81***	0.92***	0.99***				
SISf	0.89***	0.88***	0.86***	0.85***			
RSSf	0.81***	0.92***	0.94***	0.93***	0.84***		
CFUf	0.81***	0.89***	0.98***	0.96***	0.81***	0.92***	
CFUIf	0.82***	0.90***	0.98***	0.96***	0.82***	0.92***	1.00***

*SISs *Seedling infection score (screenhouse), *RSSs *Root and stem severity (screenhouse), *CFUs *Colony forming unit (screenhouse), *CFUIs *Colony forming unit index (screenhouse), *SISf *Seedling infection score (field), *RSSf *Root and stem severity (field), *CFUf *Colony forming unit (field), *CFUIf *Colony forming unit index (field)

***p = 0.001; no asterisk: p > 0.05

There were significant differences (*p* < 0.05) in days to first flowering, days to 50% flowering, days to first pod maturity, days to 95% pod maturity, pod weight, seed weight, biomass dry weight and harvest index of the genotypes evaluated under field conditions (Table 7).

**Table 7:** Yield and yield related characteristics of cowpea genotypes evaluated under field conditions

Cowpea genotype	DFF	D50 F	D1PM	D95PM	PWKg/ha	GYKg/ha	HSW	BDWKg/ha	HI
IT07K-303-1	43.33	48.33	62.33	73.00	3033	2378	18.67	3378	0.4118
IT10K-817-3	43.67	47.67	61.00	71.33	1656	1147	14.00	2267	0.3359
IT10K-837-1	43.33	48.33	63.67	74.00	1887	1410	22.67	2089	0.3500
IT11K-61-82	44.00	49.00	60.67	68.67	2040	1413	14.67	2044	0.4091
IT13K-1070-2	42.33	59.00	62.00	72.33	1718	1422	15.00	3067	0.3163
IT13K-1144-2	46.00	51.67	65.33	76.67	1149	760.0	15.33	2622	0.2238
IT14K-1424-12	49.33	55.00	68.33	74.67	900	622.0	19.00	2711	0.1870
IT14K-1682-3	51.33	56.00	67.00	73.33	1933	1529	13.33	2578	0.3686
IT14K-1850-2	50.67	58.00	70.67	81.67	771	564.0	14.00	1778	0.2410
IT14K-2030-2	43.67	48.00	63.67	71.33	1544	1084	23.33	2222	0.3281
IT14K-2113-4	45.67	55.33	65.00	78.67	651	462.0	16.33	2622	0.1483
IT84S-2049	47.00	59.33	62.67	75.00	1693	1260	17.67	2667	0.3293
KVX404-8-1x693-2	44.00	50.33	68.33	73.67	2678	1153	17.67	3644	0.2394
SARI-2-50-80	40.00	46.00	58.67	67.33	1322	933.0	15.33	2267	0.2815
SARI-3-11-100	42.00	47.67	58.33	68.00	2833	2309	22.00	3378	0.4061
SARI-3-11-80	43.33	51.33	60.67	69.67	622	407.0	15.67	3289	0.1100
SARI-6-2-6	43.33	47.67	59.67	70.00	1533	1142	20.33	3244	0.2597
Padi-tuya	45.33	50.67	64.33	71.67	838	660.0	18.33	1956	0.2522
Apaagbala	44.67	48.33	61.00	68.67	1913	1247	14.00	2489	0.3330
Songotra	42.33	48.00	60.67	72.67	856	611.0	17.33	1822	0.2506
IT97K-499-35	46.67	51.67	64.33	75.33	569	424.0	16.33	2178	0.1628
LSD (0.05)	2.10	2.73	2.32	2.274	459	332.0	1.60	316.1	0.0677
CV (%)	2.80	3.20	2.20	1.90	18.20	18.80	5.60	7.40	14.60

*DFF *Days to first flowering, *D50 F *Days 50% flowering, *D1PM *Days to first pod maturity, *D95PM *Days to 95% maturity, *PY *Pod yield, *GY *Grain yield, *BDW *Dry biomass weight, *HI *Harvest index, *HSW *Hundred seed weight

Among the genotypes classified as moderately resistant to resistant, IT11K-61-82, IT10K-837-1, SARI-3-11-100, and IT84S-2049 had days to first flowering ranging from 42.00 to 47.00 days, and days to 95% pod maturity between 68.00 to 75.00 days.

Genotypes classified as moderately resistant to resistant also had high grain yields ranging from 1,260 kg/ha (IT84S-2049) to 2,309 kg/ha (SARI-3-11-100), even under the pathogen pressure. On the other hand, susceptible genotypes such as IT14K-2030-2 and IT14K-2113-4 exhibited higher disease infection, which reflected in their lower grain yields of 1,084 kg/ha and 462 kg/ha, respectively. Interestingly, some genotypes such as IT07K-303-1, IT14K-1682-3, and IT13K-1070-2 although classified as moderately susceptible**,** maintained appreciable yields inspite of a higher disease incidence and severity. IT07K-303-1 had the highest grain yield among all genotypes, producing 2,378 kg/ha, followed by IT14 K-1682-3 with 1,529 kg/ha, and IT13K-1070-2 with 1,422 kg/ha. These genotypes can be considered to be tolerant. The remaining genotypes categorized as moderately susceptible, showed some high levels of DI, RSS, CFUI, and grain yield. For example, Padi-tuya and SARI-2-50-80 produced grain yields of 660 kg/ha and 933 kg/ha, respectively, indicating their moderate susceptibility to charcoal rot disease.

Agronomic traits were influenced by disease parameters (Table 8). DI had a significant positive correlation with SIS, RSS and CFUI. However, DI showed significant negative correlation with percentage germination, HI and SWKg/ha. SIS had a significant negative correlation with percent germination, PWKg/ha, SWKg/ha and HI. Also, RSS had a significant negative correlation with percent germination, PWkgha, and HI. Again, CFUI had a significant negative correlation with percent germination, PWKg/ha, SWKg/ha and HI.

**Table 8:** Coefficient of correlation among agronomic and yield traits and disease-related indicators

	% germination	DI	DFF	D50 F	D1PM	D95M	NO_POD.P	PWKg/ha	GYKg/ha	HSW	BDWKg/ha	HI	RSS	CFU	CFUI
DI	−0.76***														
DFF	−0.12	0.04													
D50 F	−0.14	0.01	0.58***												
D1PM	−0.33**	0.17	0.72***	0.55***											
D95M	−0.32*	0.15	0.62***	0.62***	0.78***										
NO_POD.P	0.17	−0.05	0.09	0.16	−0.03	0.04									
PWKg/ha	0.56***	−0.39**	−0.26*	−0.25*	−0.2	−0.35**	0.25*								
GYKg/ha	0.53***	−0.38**	−0.23	−0.17	−0.32**	−0.38**	0.30*	0.92***							
HSW	0.12	−0.24	−0.43***	−0.35**	−0.28*	−0.17	−0.07	0.29*	0.26*						
BDWKg/ha	0	0.04	−0.19	0.01	−0.13	−0.2	0.30*	0.47***	0.43***	0.11					
HI	0.62***	−0.46***	−0.17	−0.19	−0.29*	−0.36**	0.2	0.81***	0.86***	0.22	−0.03				
RSS	−0.67***	0.57***	−0.01	0.11	0.22	0.38**	−0.01	−0.40**	−0.35**	−0.07	0.02	−0.49***			
CFU	−0.68***	0.65***	0.13	0.09	0.30*	0.36**	−0.2	−0.43***	−0.40**	−0.15	−0.13	−0.46***	0.84***		
CFUI	−0.68***	0.65***	0.12	0.09	0.31*	0.36**	−0.19	−0.42***	−0.40**	−0.15	−0.13	−0.46***	0.84***	1.00***	
SIS	−0.97***	0.75***	0.09	0.12	0.32*	0.32*	−0.18	−0.53***	−0.50***	−0.11	−0.02	−0.59***	0.70***	0.71***	0.72***

Disease parameters: SIS, RSS and CFUI

*Significant (*p*: 0.05), **highly significant (*p*: 0.01), ***highly significant (*p*: 0.001), no asterisk: *p* > 0.05 (no significant)

## Discussion

Improved cowpea genotypes that are high yielding and also resistant to *M. phaseolina* are of prime importance to prevent high yield losses caused by *M. phaseolina* infection. In the quest to search for improved cowpea genotypes that are high yielding and also resistant to *M. phaseolina* infection, 23 cowpea genotypes pre-selected based on their high yields were evaluated in this study. Genotypes responded differently to seedling infection (SI), disease incidence (DI), root and stem severity (RSS) and colony forming unit index (CFUI) in both screenhouse and field trials. Out of the 23 genotypes evaluated excluding the checks (Apagbaala, Songotra, IT97K-499-35, Suvita-2 and IT99K-573-1-1), four were classified as moderately resistant to resistant (IT11K-61-82, IT10K-837-1, SARI-3-11-100 and IT84S-2049) three were moderately susceptible (IT07K-303-1, IT14K-1682-3 and IT13K-1070-2), two were susceptible (IT14K-2030-2 and IT14K-2113-4) while the remaining were moderately susceptible.

The disease parameters SI, DI, RSS, and CFUI were integral in classifying the genotypes. Genotypes classified as resistant to moderately resistant, such as IT11K-61-82, IT10K-837-1, SARI-3-11-100, and IT84S-2049, exhibited lower values of SI, DI, RSS, and CFUI, indicating their robust defense mechanisms against the pathogen. On the other hand, the susceptible genotypes, IT14K-2030-2 and IT14K-2113-4, and moderately susceptible genotypes were characterized by higher values of disease parameters which corresponded with more severe disease symptoms. These genotypes probably lacked effective resistance mechanisms, making them more vulnerable to infection and disease progression. This high disease parameter values underline their inability to either prevent pathogen entry or effectively respond to infection, leading to more extensive damage. Genotypes classified as moderately susceptible, including IT07K-303-1, IT14K-1682-3, and IT13K-1070-2, produced appreciable yields despite high disease incidence, seedling infection, root and stem severity and colony forming unit index of *M. phaseolina*. In a study aimed at determining the reactions of 24 bean genotypes to *M. phaseolina* infection, KMF-11-30 and ARSLAN genotypes exhibited tolerance, while 20-BBarBVD-10 genotype showed resistance [12]. This tolerance suggests that these genotypes may possess mechanisms that mitigate the effects of the pathogen, such as efficient repair of infected tissues or effective management of the pathogen's spread within the plant.

The strong positive correlation between disease parameters (SIS, RSS and CFUI) scored on cowpea genotypes in the screen house and under field conditions suggests that any of these parameters could be used in delineating resistance to *M. phaseolina*. Ouédraogo et al. [31] used only SIS to rate cowpea genotypes based on resistance or susceptibility at seedling stage. Mengistu et al. [24] and Hill [18] used RSS and CFUI. The high correlation coefficients between RSS and CFUI than with other disease parameters could be due to their indicator of severity being dependent on the degree of microsclerotial infection [24].

The most frequent genotype rank (FGR) was generated by recording the highest occuring genotype rank of SIS, RSS and CFUI from field and screenhouse experiments. Based on these, Apaagbala (resistant check) and IT10K-837-1 was classified as resistant while IT11K-61-82, SARI-3-11-100, and IT84S-2049 were moderately resistant. IT14K-2030-2, IT14K-2113-4 and the susceptible checks, IT97K-499-35 and Songotra were grouped as susceptible while the remaining genotypes were moderately susceptible. Individual genotype ranking based on only SIS, RSS and CFUI also almost followed the pattern above. FGR of IT10K-837 was resistant under screen house condition and moderately resistant under field conditions. Similarly, FGR of IT14K-1424-12 was moderately susceptible under screenhouse conditions while it was susceptible under field conditions. These give an indication of higher disease expression of disease on the field than in pot experiment under screenhouse conditions. This high expression of disease on the field could be attributed to reintroduction of inoculum, as well as synergistic effect of *M. phaseolina* with other soil-borne pathogens. Pathogens such as *Sclerotium rolfsii* was found in association with *Macrophomina* isolates from Manga, while *Rhizoctonia solani* was associated with *M. phaseolina* isolates from Akomadan. Also, root-knot nematode symptoms were observed on Charcoal rot infected cowpea at Damongo (Tengey et al., unpublished data). These show that in the field these organisms associate with *M. phaseolina* to cause severe disease.

Disease incidence (DI) was determined during field screening and was also found to have a strong and significant positive correlation with field SIS, RSS and CFUI, but a strong significant negative correlation (*r* = −0.97, *p* = 0.001) with percentage germination (plant stand). Apagbaala, IT84S-2049, IT10K-837-1, IT11K-61-82, SARI-3-11-100 have percent DI less than 10% and a high plant stand ranging from 63.33–76.67% while the remaining genotypes had DI as high as 39.33% for IT10 K-817-3 and plant stand as low as 31.67% for IT97 K-499–35. The similarity in classifications suggests that resistant genotypes could also be selected using the disease incidence or plant stand. The seedling stage disease incidence obtained in this study were lower than what was reported by Ouédraogo et al. [31]. The variations in seedling emergence (plant stand) observed could be attributed to seed rot and damping off caused by *M. phaseolina* or other soil-borne pathogen. Seed rot, pre- and post- emergence damping off were observed on cowpea from *M. phaseolina* infection [26, 34]. However, results from seedling and reproductive stage screening in pots under screen house conditions, reveal that the disease was more prominent at the seedling stage causing seed rot and damping off. According to Adekunle et al. [4], seedling damping off causes the greatest losses in cowpea production, and *M. phaseolina* is often the causal agent when moisture stress is involved. This is evident in the strong negative correlation between SIS and grain yield. Saleh et al. [34] observed soybean seedling losses due to damping off caused by *M. phaseolina*. It will be therefore be important to select genotypes that succumb to this stage of infection.

Genetic variability observed for agronomic and yield traits, agrees with studies by Adewale et al. [5], who reported significant variability in days to 50% flowering, days to maturity, pod yield, grain yield and 100 seed weight of cowpea genotypes grown in *M. phaseolina* hotspot.

The genotypes that showed resistance based on FGR of both screen house and field conditions could be considered as genotypes that are resistant to *M. phaseolina*.

The highly significant negative correlation between disease parameters (SIS, RSS and CFUI) and yield parameters (grain yield, pod yield and harvest index) suggests that the disease had an impact on yield. As disease severity increases, yield decreases. SIS had a strong negative correlation with grain yield emphasizing the devastating effect of the seedling stage infection on grain yield.

Genotypic variabilities in CFUI and RSS gives an indication of differences in the presence of sclerotia populations in the host plants’ roots and stems. This difference in compatibility with *M. phaseolina* may be due to a mix of genetic, physiological, and environmental factors as reported by Gupta et al. [16].

Apart from genetic factors, environmental factors such as drought stress causes plant tissues to weaken and allows space for microsclerotia to infect the internal plant structure blocking xylem vessels and causing plants to wilt [22, 43]. If environmental stressors are not present, *M. phaseolina* may persist in plant tissues without showing any symptoms of infection even in highly inoculated environments [6]. In this present study field experiments were conducted in the dry season with controlled irrigation to ensure the pathogen thrives and causes the expected disease symptoms. This is important because factors such as drought stress, high temperatures and age of plant have been mentioned by Afouda et al. [6] to be involved in the growth of *M. phaseolina*.

Genotypes which have been classified as resistant and moderately resistant is due to their genetic make-up. The level of resistance and susceptibility exhibited by the genotypes for the disease indicators suggest that cowpea possesses variant genes in response to *M. phaseolina* infections. The genotypes that showed resistance could indicate some resistant trait to *M. phaseolina* therefore making them non-host. On the other hand, the genotypes that showed level of susceptibility might have some susceptible trait to *M. phaseolina* therefore making them a host. Mayek-Perez et al. [22] investigated the mechanisms underlying common bean resistance to *M. phaseolina* and found that cultivars with higher water and turgor potentials were more resistant to *M. phaseolina* than susceptible cultivars. As a result, cultivars that are resistant to drought stress can also be resistant to *M. phaseolina*, and vice versa. Among the *Macrophomina* resistant genotypes, IT10K-837-1 have been reported to have resistance to drought at the seedling stage [41]. This confirms the report of Mayek-Perez et al. [22] about the relationship between a crops’ water potential or turgor potential and resistance to *M. phaseolina*. It could be interpreted that all plants that showed susceptibility had less pectin in their cell wall. According to Vorwerk et al. [42], pectin which is a plant cell wall polysaccharide is responsible for providing defense against pathogenic infection. There is, therefore, a possibility that genotypes which were susceptible in this study may have less pectin in their cell wall. French bean plant, which showed symptoms of *M. phaseolina* were observed to have 50% less pectin [27].

## Conclusion

Based on the significantly positive correlation between SIS, RSS and CFUI, any of these disease indicators can be used in screening for resistance and susceptibility of cowpea genotypes. For crop breeders who want to rapidly screen large set of cowpea genotypes, screening based on seedling infection score will be most appropriate. Of the 21 genotypes evaluated under field conditions, seven, namely IT07K-303-1, SARI-3-11-100, IT14K-1682-3, IT13K-1070-2, IT11K-61-82, IT10K-837-1 and IT84S-2049 had grain yield higher than the resistant check variety (Apagbaala). IT07K-303-1, IT14K-1682-3 and IT13K-1070-2 which were initially classified as moderately susceptible, can be considered as tolerant genotypes as they were able to withstand the infection and produced appreciable yields. The identified resistant and tolerant genotype with high grain yield and other agronomically important traits such as earliness and large seed size should be released as varieties for farmers. Genotypes that have the resistance but lack some of these agronomically important traits could be further improved through breeding before release in the future. Also genotypes that have farmer preferred characteristics such as IT14K-2030-2 but lacks resistance to *Macrophomina* could be improved upon through marker assisted backcrossing.

## Data Availability

The datasets used and/or analysed during the current study are available from the corresponding author on reasonable request.
